# Detection and Discrimination of Cotton Foreign Matter Using Push-Broom Based Hyperspectral Imaging: System Design and Capability

**DOI:** 10.1371/journal.pone.0121969

**Published:** 2015-03-20

**Authors:** Yu Jiang, Changying Li

**Affiliations:** College of Engineering, University of Georgia, Athens, Georgia, United States of America; Institute of Cotton Research of Chinese Academy of Agricultural Sciences, CHINA

## Abstract

Cotton quality, a major factor determining both cotton profitability and marketability, is affected by not only the overall quantity of but also the type of the foreign matter. Although current commercial instruments can measure the overall amount of the foreign matter, no instrument can differentiate various types of foreign matter. The goal of this study was to develop a hyperspectral imaging system to discriminate major types of foreign matter in cotton lint. A push-broom based hyperspectral imaging system with a custom-built multi-thread software was developed to acquire hyperspectral images of cotton fiber with 15 types of foreign matter commonly found in the U.S. cotton lint. A total of 450 (30 replicates for each foreign matter) foreign matter samples were cut into 1 by 1 cm2 pieces and imaged on the lint surface using reflectance mode in the spectral range from 400-1000 nm. The mean spectra of the foreign matter and lint were extracted from the user-defined region-of-interests in the hyperspectral images. The principal component analysis was performed on the mean spectra to reduce the feature dimension from the original 256 bands to the top 3 principal components. The score plots of the 3 principal components were used to examine clusterization patterns for classifying the foreign matter. These patterns were further validated by statistical tests. The experimental results showed that the mean spectra of all 15 types of cotton foreign matter were different from that of the lint. Nine types of cotton foreign matter formed distinct clusters in the score plots. Additionally, all of them were significantly different from each other at the significance level of 0.05 except brown leaf and bract. The developed hyperspectral imaging system is effective to detect and classify cotton foreign matter on the lint surface and has the potential to be implemented in commercial cotton classing offices.

## Introduction

Cotton is an important natural fiber contributing to the economy around the world. The United States is the leading cotton producing country and exporter, accounting for over one-third of global trade in raw cotton. Annually, the U.S. cotton industry provides more than $25 billion in products and services, generating about 200,000 jobs in the industry sectors from farms to textile mills [1]. In the U.S., cotton is harvested by mechanical harvesters, such as cotton pickers and strippers, with former being more widely used [2]. During the mechanical harvest process, cotton lint is mixed with a large quantity of foreign matter (FM), such as leaves, bract, stem, hull, etc., affecting the quality, profitability, and marketability of the cotton lint. Although most large-sized cotton FM (e.g. hull) can be directly removed after ginning, a large amount of FM is cut into small pieces and still remains mixed with lint. The associated FM are diverse in nature and respond differently to textile cleaning and further processing. For instance, leaves only lead a relatively higher loss of the lint during textile cleaning, whereas plastic materials can have the most harmful effect on the quality of the textile products, not only adversely affecting spinning performance, but also showing up as faults in the textile production, especially after dyeing [3]. Therefore, cotton FM detection and classification are critical to cotton and textile industry.

Currently a small cotton sample from each bale is sent to USDA classing offices to evaluate the quality using the High Volume Instrument (HVI). The HVI is widely used in order to objectively measure the quality of cotton lint properties including color, length, strength, micronaire, overall cotton FM level, leaf grade, etc. [4]. Another system, the Advanced Fiber Information System (AFIS), is also available for researchers to measure the quality of individual fiber and short fiber which cannot be evaluated by the HVI [5]. Although both the HVI and the AFIS can measure a number of quality properties, none of them have the capability to quantify and differentiate various types of cotton FM at present.

To address this issue, many researchers have explored different technologies to detect and classify various types of cotton FM. Since conventional RGB color cameras are inexpensive and relatively easy to setup, researcher have established color imaging systems to detect and identify cotton FM. Xu et al. developed an imaging system based on a color CCD camera with Xenon illumination to predict cotton FM and their color parameters [6]. The results were highly correlated to the results obtained from the HVI, indicating the potential of using color imaging systems to detect cotton FM. However, this study was only detecting the presence of the cotton FM. Yang et al. developed an automatic machine to detect and classify cotton FM using a monochrome CCD camera under UV illumination, and a color CCD camera under white light [7, 8]. A total of 9 types of foreign matter were used, including feathers, papers, polypropylene twines, chemical fibers, cloths, hemp ropes, hairs, packing materials, plastic films. The results showed that the mean accuracy of classifying cloth, feather, hair, hemp rope, and plastics was 92.34% when both color and shape features were used. However, there were limitations of that system. The classification rate significantly decreased when the color of the FM was similar to the color of the lint. For instance, the classification rate of feather was only 79.59%. In addition, the shape feature extracted from the color images might change overtime and is not a reliable feature for cotton FM classification [9].

Due to the limitations of the color imaging systems, researchers have conducted extensive research in using spectroscopy to detect and classify cotton FM in the spectral range from near infrared (NIR) to infrared (IR) because the chamical properties of the FM in these ranges are usually distinct. Fortier et al. demonstrated the procedure of using the NIR spectroscopy to recognize seed coat, hull, leaf, and stem from cotton lint [10]. Although the average classification rate was 94.3% for the prediction set, the classification accuracy of stem was only 77.8% due to its nonuniform composition. That was because the spectroscopic methods are point-based, and the spectral responses could change significantly when measuring at different positions on a nonuniform sample. Himmelsbach et al. proposed to use a Fourier-Transform Mid-Infrared (FT-MIR) spectrometer based system to identify 5 types of cotton FM (leaf, hull, bract, stem outer, and seed coat outer) by comparing the spectra between the samples and the standards in the FM database [11]. Although the results showed that even the lowest classification rate was more than 90%, the spectra references had to be continually updated until it included all types of cotton plant from multiple crop years and different varieties. Thus, this requirement highly limited the system. Allen et al. also proved the potential of using FT-IR spectroscopy to classify cotton FM, but the results were affected by both FM uniformity and sample preparation [12]. For instance, the spectra of FM samples obviously changed when the FM was nonuniform or the FM irreversibly lost chemical components by heating process. Although all three spectroscopy based methods succeeded in detecting and identifying cotton FM, they were still conceptual approaches for the cotton industry because the sample preparation was quite tedious and the results sometimes varied due to different preparation procedures (e.g. with/without heating). In addition, since the spectroscopic measurements are point-based, there is no spatial information regarding the FM samples, which limits the potential of using spectroscopic methods for automated detection and classification of FM in cotton classing offices.

In addition to the aforementioned approaches, energy attenuation based methods also have been explored such as X-ray. Pai et al. used X-ray micro tomography to recognize 3 types of cotton contaminants including seed-coat fragments, bark, and polypropylene [13]. The average classification rate of 96% was achieved but this method was too time consuming to be applicable to the industry. Thus, Dogan et al. from the same lab improved the X-ray method in real time [14]. Although the results showed that X-ray was successful to recognize cotton FM based on the difference in attenuated energy among various FM, this modality was limited by its high cost and risk of radiation accidents.

Recently, the hyperspectral imaging technique, which provides both spatial and spectral information, has been widely used for quality assessment of agricultural products [15]. To the best of our knowledge, Guo et al. is the only group who used hyperspectral imaging (HSI) technique for cotton FM classification in China [16]. A total of 11 types of foreign matter were divided into four groups: colored (e.g. black hair), light color (e.g. gray woven bag), white (e.g. white hair), and colorless (e.g transparent plastics). The results showed the recognition rate of gray polypropylene fiber and black hairs were up to 96.8%, and the rate of white polypropylene fibers were more than 80%. However, there are two major limitations of Guo’s study. Firstly, the FM samples used in Guo’s study usually had different surface color, while the FM found in the U.S. cotton typically had similar surface color. This limited the potential of HSI system only in classification of the cotton FM found in the Chinese cotton and the FM with different surface color. In fact, compared with the color feature, the spectral response could provide additional information for classifying the FM with similar color. Secondly, the system is based on commercial software, which limits further expansion of the HSI system, such as adding image processing functions for specific applications.

The main goal of this paper was to explore the potential of detecting and classifying cotton FM commonly found in the U.S. cotton using hyperspectral images. The specific objectives were to: (i) develop a custom-built software for HSI system control and image acquisition, (ii) acquire hyperspectral images of 15 cotton foreign materials usually found in the U.S. cotton and extract their mean spectra, (iii) explore the potential of detecting and classifying these FM by using principal component analysis (PCA) and statistical tests.

## Hyperspectral Imaging System

### Platform Integration

**Fig 1:**
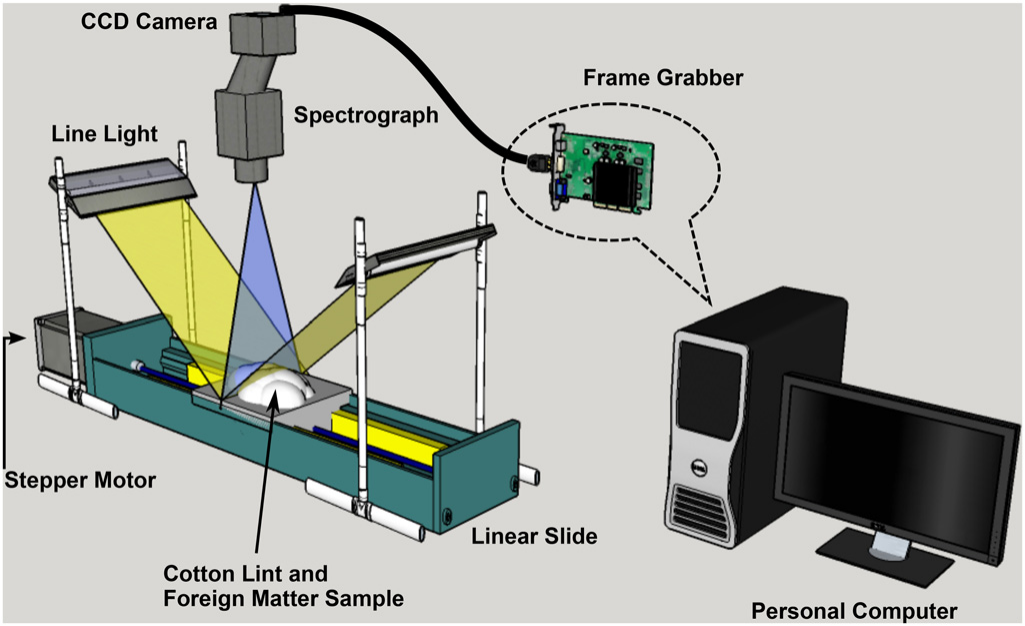
The schematic of the system.

A push-broom based HSI imaging system, including both hardware and software components, was developed at the Bio-sensing and Instrumentation Laboratory at the University of Georgia. The hardware platform consisted of a hyperspectral imaging unit, an illumination unit, and a motorized linear slider (Fig. 1), all installed in an enclosed box to avoid the influence of the ambient light. The imaging unit consisted of an optical lens (F/1.4, f = 17.53mm, XNP 14/17-0503B, Schneider Optics, Hauppage, NY, USA), a 14-bit CCD camera with 1392 × 1040 pixel resolution (ICL-B1410, Imperx Inc., Boca Raton, FL, USA), and a prism-grating-prism imaging spectrograph (ImSpector V10E, Specim, Oulu, Finland). The spectral range of the spectrograph is from 400 nm to 1000 nm. The unit was linked to a PC (Dell Inc., Austin, TX, USA) through a frame grabber (NI PCI-1426, National Instruments Inc., Austin, TX, USA) with standard camera link cable. The illumination unit included a 150W quartz tungsten-halogen light source (Fiber-Lite DC950, Dolan-Jenner Industries, Boxborough, Mass., USA) shedding light onto the cotton samples through a bifurcated line light guide. The light source worked under direct current (DC) regulated mode with its maximum intensity. A sample holder, a stepper motor (MDrive 23 Plus, Schneider Electric Motion, USA) and a liner slider (MS33, Thomson Industries Inc., Wood Dale, IL, USA) were integrated to move samples under the line scan imaging system. The motor was connected with the computer via a RS-422-to-USB convertor (CP210X, Silicon Laboratories Inc., Austin, TX, USA).

### Software Design

**Fig 2:**
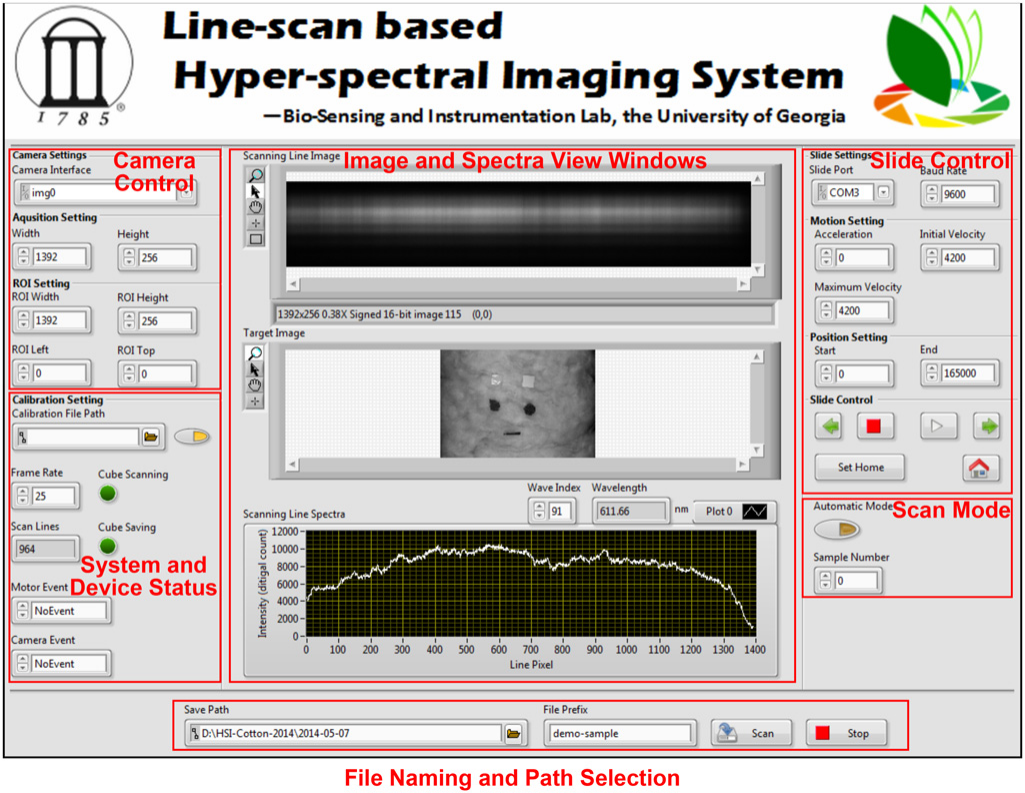
The front panel of the custom software.

**Fig 3:**
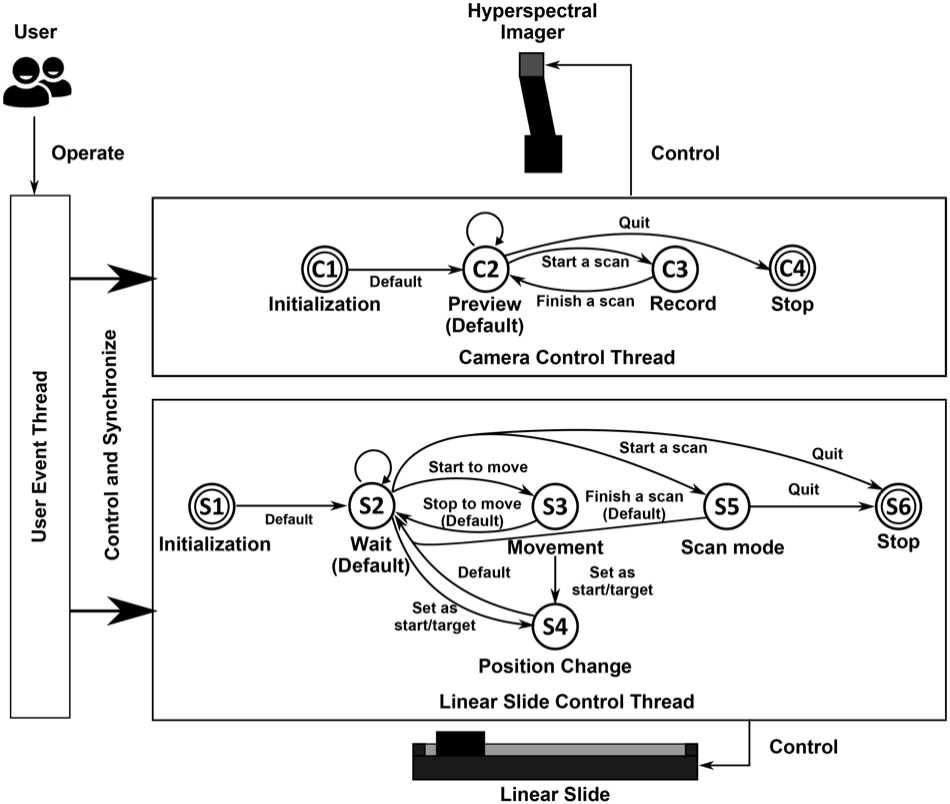
The software architecture and the state diagram of the event-driven finite state machine.

A custom software of image acquisition and system control was developed using LabVIEW 2012 (National Instruments Inc., Austin, TX, USA). The front panel consisted of six subareas for system configuration and image acquisition (Fig. 2). The software was based on multi-thread structure and event-driven finite state machine (EFSM). A total of three threads were established for controlling camera and slide and responding user requests, respectively. The motor and camera control threads were designed based on the EFSM model, and each of them had a set of finite states (Fig. 3). The camera had four states, including device initialization, preview, record, and stop, whereas the linear slider had six states, namely, initialization, wait, movement, position change, scan, and stop. The two devices worked independently and were only on one of their states at a time. In order to complete specific tasks, the state transition of the two devices was driven by certain user events.

All user interactions were processed and responded by the user-event thread, and thus synchronously changing the work state of the motor and slide. For instance, when image acquisition was requested, the camera became record mode and the linear slide turned into scan state simultaneously. Compared to commercial software, the developed software can be easily extended and customized. For example, it is easy to integrate image processing functions in the software, or to install and control extra devices integrated in the system.

Currently, the software scans the sample line by line, and then reconstructs these scanned lines to a hyper-spectral cube. Prior to scanning, the frame rate of the camera and the speed of linear slide are set by users according to the requirement of spatial resolution. During scanning, the top window of the “Image and Spectra View” shows the image of one scan line where the horizontal direction is spatial dimension and the vertical direction represents spectral dimension. Meanwhile, the bottom window plots the reflectance intensity along the scan line at a certain wavelength set by the user. After scanning, the window in the middle shows the whole spatial image of the sample at the set wavelength. The hyper-spectral cube of the sample will be automatically named and stored using the pre-defined filename prefix and saving path. In addition, a batch mode is provided for image acquisition of multiple samples. In this mode, there is a time interval of 30 seconds for placing sample on the target holder, and subsequently the system will automatically start the scan based on the previous configuration.

## Materials and Methods

### Ethics Statement

The samples, including cotton lint, inner and outer bark, inner and outer stem, brown leaf, bract, hull, and twine, seed coat inner, seed coat outer, seed, green leaf, and plastic bale packaging, were harvested and provided by the Microgin at the University of Georgia, Tifton campus (31°29′42.6″N, 83°31’42.2″W). The study was carried out on private land and the owner of the land gave permission to conduct the study on this site. Furthermore, the field studies did not involve endangered or protected species.

### Cotton Trash and Lint Samples

**Fig 4:**
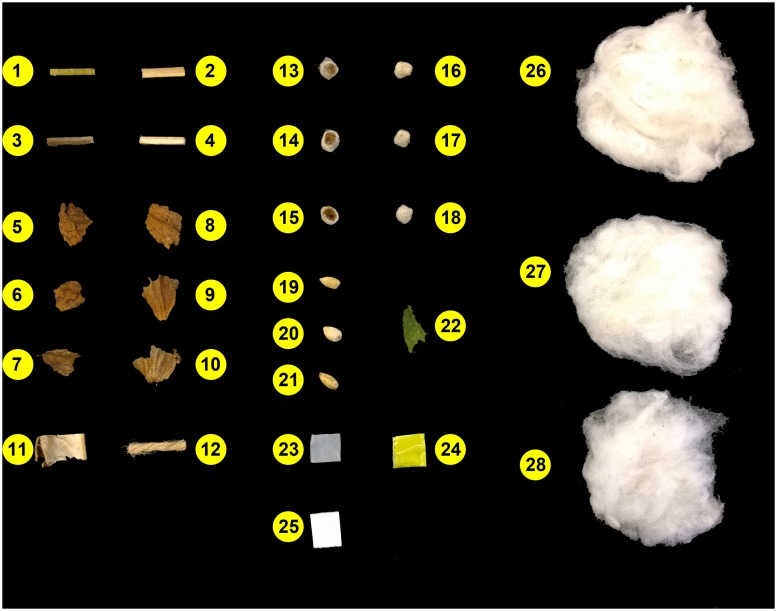
Cotton foreign matter and cotton lint samples used in this paper. 1-bark outer, 2-bark inner, 3-stem outer, 4-stem inner, 5, 6, and 7-brown leaf from DP 1050, NG5315, and PHY 339 respectively, 8, 9, and 10-bract from DP 1050, NG5315, and PHY 339 respectively, 11-hull, 12-twine, 13, 14, and 15-seed coat inner from DP 1050, NG5315, and PHY 339 respectively, 16, 17, and 18-seed coat outer from DP 1050, NG5315, and PHY 339 respectively, 19, 20, and 21-seed from DP 1050, NG5315, and PHY 339 respectively, 22-green leaf, 23-plastic bag, 24-plastic bale packaging, 25-paper, and 26, 27, and 28-cotton lint of DP 1050, NG5315, and PHY 339 respectively.

A total of 15 types of cotton foreign matter samples were collected and analyzed. To facilitate the discussion, the 15 types of cotton FM were divided into two groups based on the similarity of their surface color. The first group includes inner and outer bark, inner and outer stem, brown leaf, bract, hull, and twine, and the second contains seed coat inner, seed coat outer, seed, green leaf, plastic bag, plastic bale packaging, and paper (Fig. 4). Brown leaf, bract, seed coat inner, seed coat outer, and seed were collected from seed cotton of three cultivars harvested during the Fall 2013 harvest season: Delta Pine (DP) 1050, NexGen (NG) 5315, and PHY 339, while inner and outer bark, inner and outer stem, hull, and green leaf were collected from seed cotton of Delta Pine 1050. Paper (Boise X-9 Copy Paper, Model No. OX9001) and twine (Lehigh Groupp 530 Jute Twine, Model No. 016033) were purchased from a local store (Walmart, 33°56′02.3″N, 83°18’29.9″W), while plastic bag and plastic bale packaging were obtained from the microgin in Tifton, Georgia, USA (31°29′42.6″N, 83°31’42.2″W). All samples were stored in enclosed paper bags at room temperature (23 ^∘^C) for five months before the experiment. Subsequently for each type of samples, 30 replicates were randomly collected and cut into ∼ 1 cm^2^ pieces with scissors. Prior to imaging, all were stored in sealed plastic containers at room Individual replicates were placed on top of cotton lint for imaging.

#### System calibration

Prior to acquiring images, the system was calibrated in the spatial and spectral dimensions using the method proposed by Lawrence [17]. The spatial resolution was calculated by using the optical test pattern (USAF Resolution Target Pocket Size, Edmund Optics Inc, Barrington, NJ, USA). In order to get more accurate regression in spectral dimension, three calibration lamps were used, including a Krypton lamp (Model 6031, Oriel Instruments, Stratford, CT, USA), a Xenon lamp (Model 6033, Oriel Instruments, Stratford, CT, USA), a Hg (Ar) lamp (Model 6035, Oriel Instruments, Stratford, CT, USA).

#### Image Acquisition, Calibration and Region of Interest (ROI) Selection

**Fig 5:**
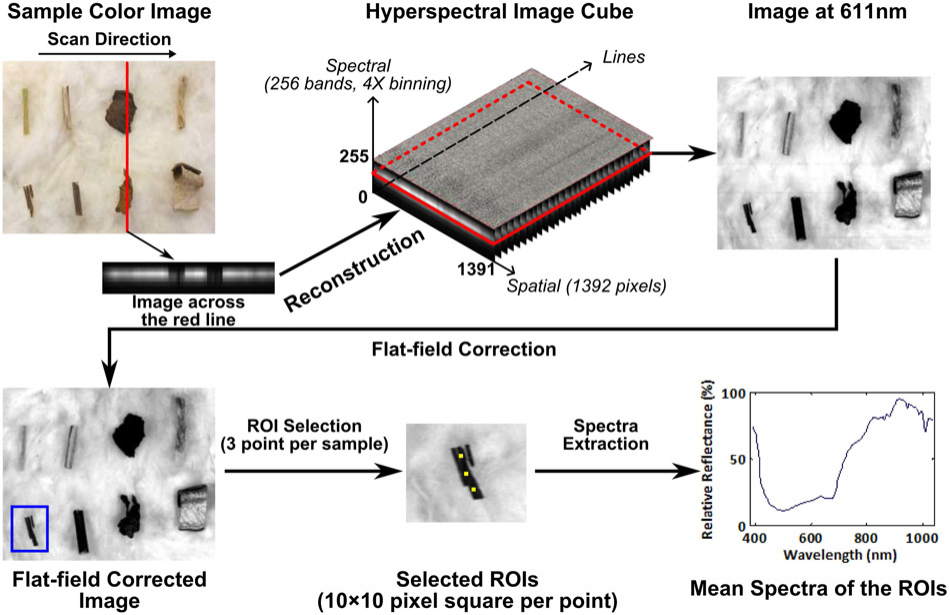
The procedure of image acquisition, calibration, ROI selection and spectra extraction.

All images were acquired using reflectance mode which obtained the reflected light intensity of the sample surface (Fig. 5). The scan speed was 2.67 mm/s at 25 frames per second (FPS). Raw images were binned 4x in the spectral dimension, with the resulting resolution of one line image of 1392 pixels in spatial dimension and 256 pixels in spectral dimension. Prior to collecting sample images, an image of a 99% reflective panel (SRT-99-050, Labsphere Inc., North Sutton, NH, USA) was obtained as the white reference, and dark reference images were acquired by covering the optical lens. All sample images were calibrated using flat field correction to remove the artifacts from images caused by nonuniform illumination or variations in the pixel-to-pixel sensitivity of the detector.

In order to extract spectra from images of cotton FM on top of lint, three ROIs were selected for each sample using a semi-automatic method. First, three points were manually determined in the image as central points of three ROIs. Typically, the ROI points should be in center of the sample to avoid edge effects. Then, all the image processing was performed using MATLAB (MATLAB R2013b, The MathWorks, Inc., Natick, MA). Three 10×10 pixel squares were automatically generated based on the selected points as the ROIs to extract the spectral information of each sample. Therefore, a total of 300 pixels were averaged to represent each sample in the image.

### Principal Component Analysis (PCA) and Statistical Test

Principal Component Analysis (PCA) is a statistical tool that represents data using uncorrelated variables named principal components (PC) instead of original variables which are possibly correlated. The total number of PCs is less than or equal to the number of original variables, however, only the PCs with high eigenvalues are considered as the most contributing PCs. The PCA was performed using 256 bands in MATLAB, and the results showed that PCs with the top three highest eigenvalues represent more than 99% of the variation of the dataset. Therefore, the top three PCs were selected as the important PCs to describe cotton FM. The clusterization patterns in the PC score plots were examined to show the capability of detecting and identifying of different types of cotton FM.

To further explore the potential of identification of FM using PCA, multivariate analysis of variance (MANOVA) tests were performed using the first three PCs in SAS (SAS 9.3, SAS Institue Inc., Cary, NC, USA). All three PCs were considered as multiple variables for each treatment when MANOVA tests (Hotelling paired-test) were conducted with a significance level of 0.05.

## Results and Discussion

### Calibration in Spectral Dimension

The calibration results showed that there was no “keystone” or “smile” distortion in the spatial and spectral dimensions, and thus no requirement of correction on distortion was needed. The spatial resolution was 1.26 line-pairs per micrometer. Seventeen representative wavelengths from 435.84 nm to 895.2 nm were identified and used to establish the regression equation between pixels in the spectral dimension and wavelengths. The errors between the standard wavelengths and calibrated wavelengths were less than 1 nm except the pixel number 82 and 177 (Table 1). All the errors are acceptable because the spectrograph has a nominal spectral resolution of 2.8 nm.

**Table 1:** Calibration of the spectral accuracy using the standard spectra from Krypton, Xenon, and Hg(Ar) lamp.

Pixel Number (256 in total)	Standard Wavelength (nm)	Calibrated Wavelength (nm)	Error (nm)
19	435.84 (Hg (Ar))	435.04	-0.80
65	546.07 (Hg (Ar))	546.88	0.81
69	557 (Krypton)	556.71	-0.29
78	579.07 (Hg (Ar))	578.89	-0.18
82	587.1 (Krypton)	588.77	1.67
150	760.19 (Krypton)	759.38	-0.81
154	769.45 (Krypton)	769.57	0.12
160	785.48 (Krypton)	784.88	-0.60
170	810.44 (Krypton)	810.48	0.04
173	819 (Krypton)	818.18	-0.82
175	823.2 (Xenon)	823.32	0.12
177	829.81 (Krypton)	828.46	-1.35
186	850.9 (Krypton)	851.66	0.76
196	877.7 (Krypton)	877.54	-0.16
198	881.9 (Xenon)	882.73	0.83
202	892.9 (Krypton)	893.11	0.21
203	895.2 (Xenon)	895.71	0.51

### Hyperspectral Reflectance Images

**Fig 6:**
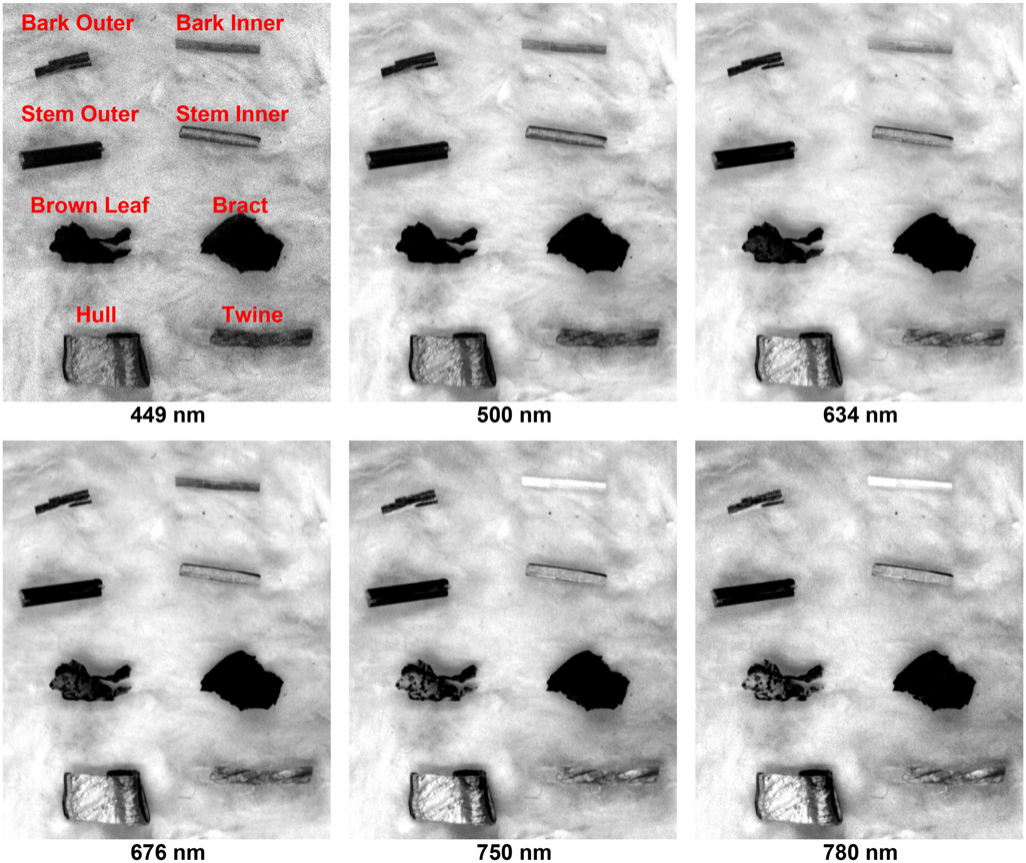
Single band images of eight brown trash at six representative wavelengths.

**Fig 7:**
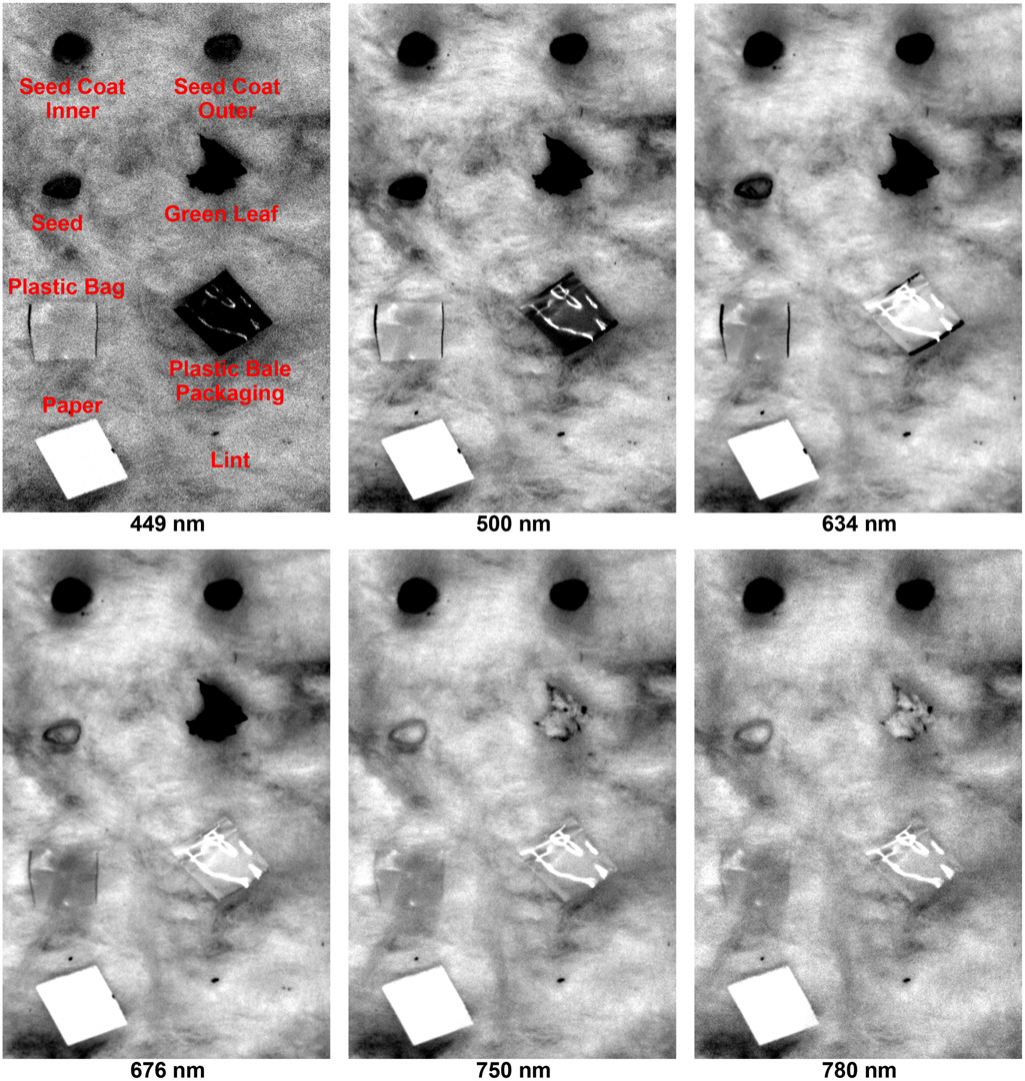
Single band images of seven non-brown trash and lint at six representative wavelengths.

Single-band reflectance images of cotton foreign matter were selected at 6 representative wavelengths from 449 nm to 780 nm (Fig. 6 and Fig. 7). In general, the quality of the images was fairly good for detection and recognition of cotton FM on the lint surface. However, the signal-to-noise ratio (SNR) of the images at 449 nm was relatively lower than at other wavelengths due to the low quantum efficiency of the camera at this wavelength. In addition to the SNR of the camera, the uneven surface of the cotton lint also significantly affected the quality of images of cotton lint (compare the lint area between Fig. 6 and Fig. 7). Although other wavelengths were not shown for the brevity reason, some lint areas looked dark in the whole spectral range. These dark areas were due to the tangled or rugged surface and sometimes were difficult to be differentiated from the dark areas of the real FM samples (e.g. the lint area in Fig. 7). Because of this challenge, the detection and identification of cotton FM could not be performed at the pixel-level in the image. Therefore, the mean spectra of samples were extracted using the ROI method instead of the automated masking to obtain the spectral information.

Overall, the reflectance difference between cotton lint and all FM (except plastic bag) was clearly observed at visible range (449 nm to 750 nm). Most cotton FM appeared darker than the lint because of their high absorption of light, while paper looked brighter due to its high reflectance of light. It was noteworthy that the contrast between the plastic bag and lint was lower than the contrast between other FM, especially at longer wavelengths (750 nm and 780 nm). This was because the plastic bag used in the present research was transparent without any pigment, so only little light was absorbed by the bag piece. Most light was either directly reflected by the plastic piece or transmitted and reflected by the lint, and received by the CCD camera. For other cotton FM, they also showed different patterns and trends in the images. Most FM in the first group (e.g. bark inner, stem inner, brown leaf, hull, and twine) became brighter at longer wavelengths, and the same pattern was observed for cotton seed, green leaf, and plastic bale packaging in the second group as well. Bark outer, stem outer, and seed coat inner and outer appeared dark in all 6 images. In contrast, paper and plastic bag were always bright. It was also observed that some FM with similar surface color exhibited good contrast between each other in the near-infrared (NIR) range. For instance, bark inner was distinguished from others because it appeared brighter due to its relatively high reflectance at 750 nm and 780 nm. Therefore, the information in the NIR range could be used for identifying cotton FM with similar surface color. However, it is noteworthy that the NIR range (750 nm to 1000 nm) covered by the current system was small due to the instrumentation limitation. Therefore, longer wavelengths in the NIR range (1000 nm to 2500 nm) need to be further explored for discriminating cotton FM.

### Reflectance Spectra

**Fig 8:**
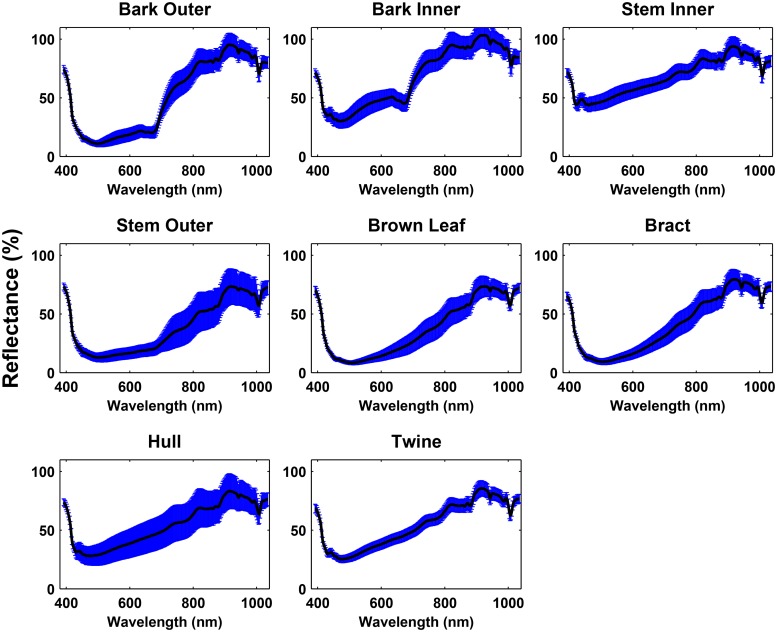
The mean spectra (black solid line) and standard deviation (error bar) of eight brown trash.

**Fig 9:**
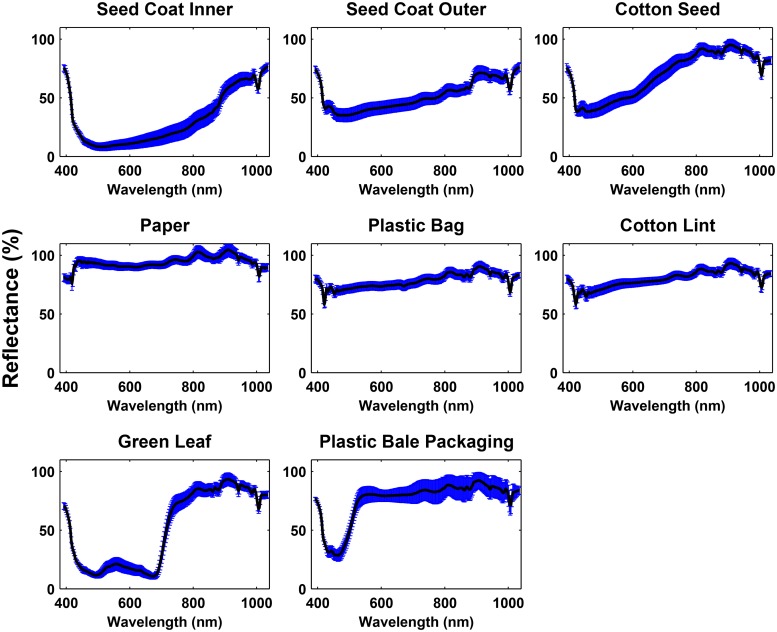
The mean spectra (black solid line) and standard deviation (error bar) of seven non-brown trash and lint.

The mean spectra of cotton lint was clearly different from that of most cotton FM except plastic bag (Fig. 8 and Fig. 9). Although the intensity of plastic bag was lower than that of lint, the difference between them was quite small due to the little absorbed light. Since plastic bag was transparent without pigment, the light shined on the plastic bag pieces was mostly reflected instead of being absorbed. The intensity of the cotton lint was higher than that of most cotton FM in the range from 400 nm to 750 nm but was lower than paper in the whole spectral range. This occurred because most cotton FM contain pigments or chemical components which absorb light in the visible range (400 nm to 750 nm), while the lint fiber is reflective in this range. However, paper was an exception because it is a highly reflective artificial material, and thus its intensity was higher than the lint fiber in the whole spectral range. Therefore, from the mean spectra perspective, all cotton FM on the lint surface could be detected.

Various cotton FM showed difference in their mean spectra as well. In the first group, the lowest reflectance intensity of all cotton FM appeared at ∼ 500 nm with the reflectance value in the range from 10% to 30% except stem inner (40% to 50%) owing to their brown surface. This occurred because the light was absorbed by the carotenoids at ∼ 500 nm [18]. However, since stem inner consisted of vascular cambium and secondary xylem [19], its color was brighter than that of other cotton FM in this group, thus the lowest reflectance of stem inner was approximately 50% (Fig. 8). Besides, a local minimum was observed at ∼ 700nm for both the inner and outer part of bark but not for others due to the absorption caused by retained chlorophyll in bark [18]. In addition, since the reflectance of bark inner is higher than that of bark outer, they could be separated as well. The stem outer showed similar spectral shape as bark outer but the reflectance was lower, because bark outer is the outermost part of stem and it contains fewer layers than stem outer which absorbs more light [19]. There was no obvious difference between brown leaf and bract. Despite similar spectral shape between the hull and twine, the reflective intensity of hull was 5% higher than that of twine in the range from 500 nm to 600 nm.

In the second group, the reflective intensity of most cotton FM was 50% higher than that in the first group because of the bright surface color (Fig. 9). Green leaf clearly demonstrated two valleys at ∼ 500 nm and ∼ 700 nm respectively due to the absorption of carotenoids and chlorophyll, but the valley at ∼ 700 nm was different from that of bark inner and outer because green leaf has more chlorophyll. There was a local minimum that appeared at 500 nm on the spectra of plastic bale packaging, which was because the yellow pigment absorbs more light. Moreover, the reflectance of plastic bale packaging was high since it was covered by a shiny surface coating (typically polymer). The reflectance of plastic bag and paper was also high and did not change with the increase of wavelengths, while seed coat inner, seed coat outer, and cotton seed became brighter at longer wavelengths. The materials of seed coat inner and outer, and cotton seed have different physical and chemical properties. For example, seed coat inner contains yellow pigment, seed coat outer is covered by lint fiber, and cotton seed mainly consists of proteins. Therefore, there were differences in reflectance at bands from ∼ 600 nm to 800 nm between these three FM.

In addition to the difference in the mean spectra, the variation of the spectra from various cotton FM also differed. The spectral variation of the non-botanical cotton FM (e.g. plastic things and twine) was smaller than that of the botanical ones (e.g bark, stem, leaf, etc.), because the surface color of these artificial matter was quite uniform than that of the botanical FM. For instance, although the mean spectra of hull and twine were similar, their spectral variation were quite different. This occurred because hull consisted of both dark and light brown parts, and the two parts had different light absorption ability, resulting in a large variation of its spectra. In contrast, twine is an artificial product whose surface color was more uniform than the botanical FM, and therefore its spectra variation was small.

### PCA and Statistic Test Results

**Fig 10:**
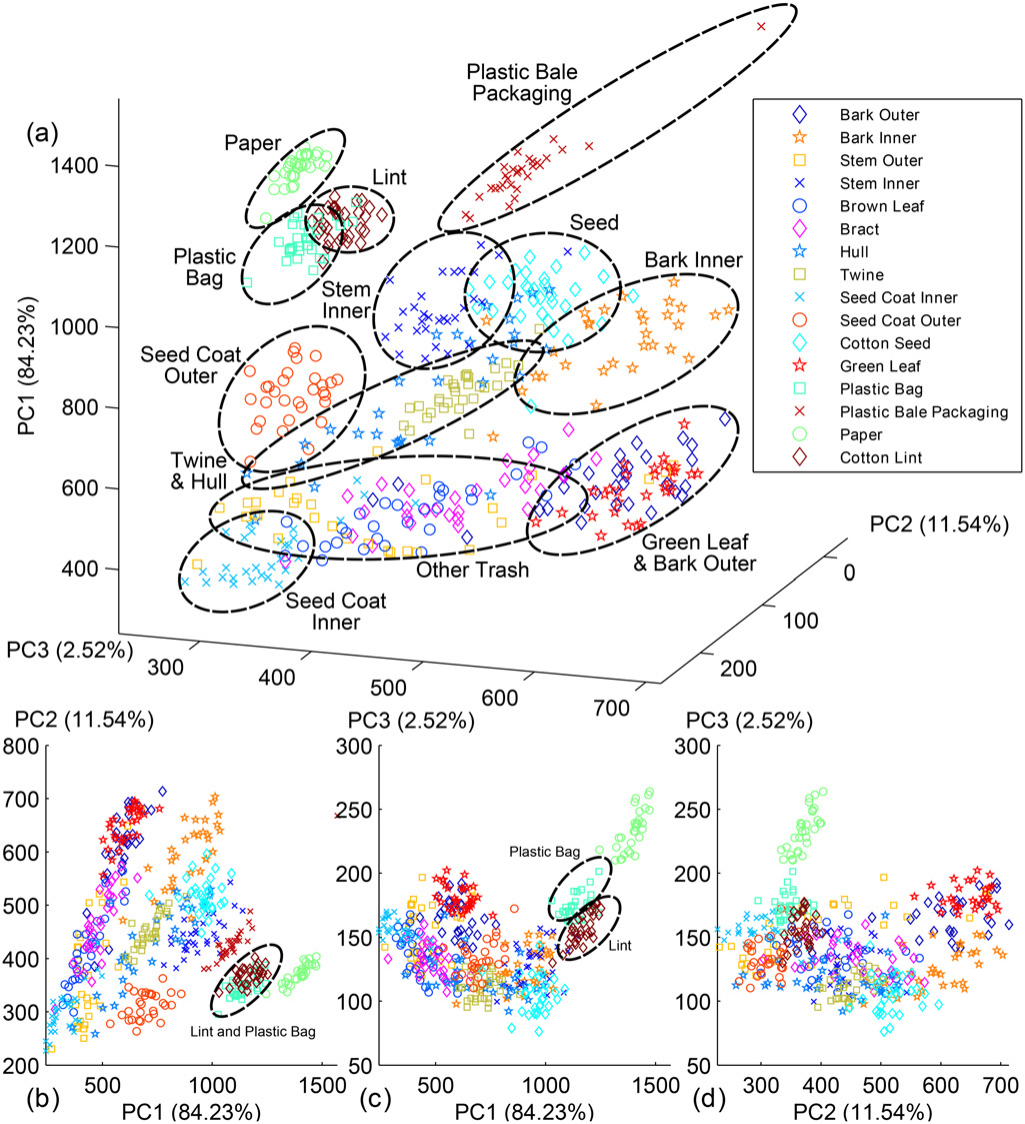
PCA score plot of 15 types of cotton trash and cotton lint. (a) clusters under the top 3 PCs space, (b), (c), and (d) are projection on PC1 vs PC2, PC1 vs PC3, and PC2 vs PC3 respectively.

To further explore the capability of the proposed system to detect and identify various cotton FM, all samples were transferred from the original spectra and plotted in the PC feature space (Fig. 10). The PCs with the top three highest eigenvalues covered the deviation of the samples at 84.23%, 11.54%, and 2.52%, respectively. Cotton lint was clearly separated from all cotton FM in the PC space, indicating the potential to detect cotton FM on top of cotton lint using hyperspectral imaging. This occurred because lint was clearly different from FM under PC1 vs PC2 except plastic bag (Fig. 10 b), however, lint was further separated from plastic bag under PC1 vs PC3 (Fig. 10 c).

A cluster was considered as unique if the cluster did not overlap with others in the PC space (from any of the combinations of the first three PCs) or its center was separated from others even with certain overlap with others. Thus, 8 out of 15 types of cotton FM formed unique clusters in the PC space, including paper, plastic bag, plastic bale packaging, seed coat inner and outer, seed, stem inner, and bark inner. More specifically, they were clearly clustered under PC1 vs PC2 (Fig. 10 b). This is mainly because all aforementioned cotton FM had unique spectral pattern and trend, resulting in separate clusters in the PC score plot. Moreover, the overall intensity of stem inner and bark inner were higher than others, and thus their clusters were separated from others. However, other cotton FM were mixed with each other: twine mixed with hull, green leaf overlapped with bark outer, and stem outer blended with brown leaf and bract. It is also noteworthy that although hull shared the similar cluster core with twine in the PC space, the variation of twine was much smaller than hull, and thus they formed a concentric circle in which twine was inside and hull was outside.

**Fig 11:**
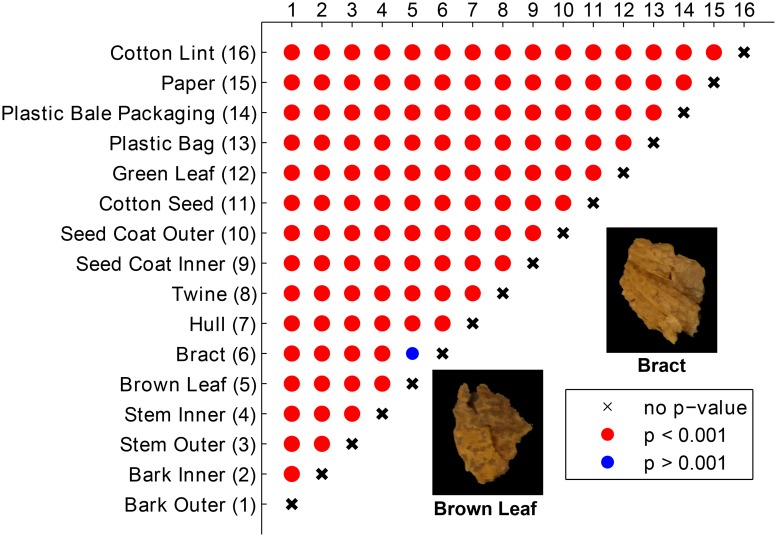
p-values of the Hotelling paired-test using the feature set of top three PC scores.

The MANOVA (Hotelling paired-test) was performed on the sample data using the feature set containing top three PCs, and the p-values of each pair were plotted as a confusion matrix (Fig. 11). When all three PCs were considered, some of the FM that overlapped in the PC score plot showed a statistical difference. Bark outer and green leaf were statistically significant because the difference in PC2 and PC3 scores contribute to the distinction. The MNOVA test revealed that hull was statistically different from twine. However, brown leaf and bract were not statistically significant. The significant difference between each individual FM showed the potential to identify them.

### Conclusion

A push-broom based HSI system and a multi-thread based software were developed to serve as a potentially effective tool to discriminate cotton FM found in the U.S. cotton lint. The visible range can be used to detect all 15 types of cotton FM on the lint surface and to differentiate the FM with different surface color, whereas the NIR range is more suitable to classify the FM with similar surface color. The mean spectra of all the FM were statistically different except for brown leaf and bract. Future studies will be focused on feature selection and classification based on the mean spectra.
